# Description of a multidisciplinary initiative to improve SCIP measures related to pre-operative antibiotic prophylaxis compliance: a single-center success story

**DOI:** 10.1186/s13037-014-0037-2

**Published:** 2014-09-17

**Authors:** Tori Sutherland, Jennifer Beloff, Marie Lightowler, Xiaoxia Liu, Luigino Nascimben, Alan D Kaye, Richard D Urman

**Affiliations:** Department of Anesthesia, Critical Care and Pain Medicine, Beth Israel Deaconess Hospital, Boston, MA USA; Center for Clinical Excellence, Brigham and Women’s Hospital, Boston, MA USA; Department of Anesthesiology, Perioperative and Pain Medicine, Brigham and Women’s Hospital/Harvard Medical School, Boston, MA 02115 USA; Chairman, Department of Anesthesiology, LSUHSC, School of Medicine, New Orleans, LA USA

**Keywords:** Patient safety, Surgical care improvement project (SCIP), Provider notification, Surgical site infection (SSI)

## Abstract

**Background:**

The Surgical Care Improvement Project (SCIP) was launched in 2005. The core prophylactic perioperative antibiotic guidelines were created due to recognition of the impact of proper perioperative prophylaxis on an estimated annual one million inpatient days and $1.6 billion in excess health care costs secondary to preventable surgical site infections (SSIs). An internal study was conducted to create low cost, standardized processes on an institutional level to improve compliance with prophylactic antibiotic administration.

**Methods:**

We assessed the impact of auditing and notifying providers of SCIP errors on overall compliance with inpatient antibiotic guidelines and on net financial gain or loss to a large tertiary center between March 1st 2010 and September 31st 2013. We hypothesized that direct physician-to-physician feedback would result in significant compliance improvements.

**Results:**

Through physician notification, our hospital was able to significantly improve SCIP compliance and emphasis on patient safety within a year of intervention implementation. The hospital earned an additional $290,612 in 2011 and $209,096 in 2012 for re-investment in patient care initiatives.

**Conclusions:**

Provider education and direct notification of SCIP prophylactic antibiotic dosing errors resulted in improved compliance with national patient improvement guidelines. There were differences between the anesthesiology and surgery department feedback responses, the latter likely attributed to diverse surgical department sub-divisions, frequent changes in resident trainees and supervising attending staff, and the comparative ability. Provider notification of guideline non-compliance should be encouraged as standard practice to improve patient safety. Also, the hospital experienced increased revenue for re-investment in patient care as a secondary result of provider notification.

## Introduction

Annually, surgical site infections (SSIs) affect 500,000-1,000,000 patients and are one of the most common types of hospital-acquired infection, accounting for 20-40% of all hospital acquired infections [1-4]. There is also a significant associated morbidity and mortality [5,6]. On average, a SSI increases a hospital stay by 9.7 days [2]. Nationally, this translates into 406,730 hospital-days and $900 million in hospital costs per year [2,7]. One analysis found that the cost of care doubles for a patient who develops a surgical site infection [8]. Because a surgical site infection can present up to thirty days post-operatively, the same analysis detected an additional 91,613 readmissions, 521,933 hospital days and $700 million in costs after the initial discharge [2]. In summary, the annual SSI burden in the United States is approximately one million inpatient days and $1.6 billion.

The Centers for Medicare and Medicaid Services (CMS) and the Centers for Disease Control and Prevention (CDC) launched the Surgical Care Improvement Project (SCIP) in 2005 to decrease perioperative complications, including surgical site infections. With regard to SSIs, the three core measures associated with decreased perioperative infection are initiating prophylactic antibiotics within one hour of surgical incision (Inf-1), administering the appropriate antibiotic (Inf-2) and discontinuing antibiotic use within twenty-four hours (Inf-3) [9]. The significance of these measures was magnified in 2011 when the Affordable Care Act incorporated Inf-1, 2 and 3 into its process of care bundle that is used to rate hospital performance and reimbursement [10,11].

Hospitals have been tasked with improving provider compliance over a short time period and are increasingly being burdened with significant financial penalties if they fail to meet government and private insurance thresholds. To date, few studies have demonstrated the role of performance feedback on patient satisfaction, health outcomes and cost of care. In the perioperative setting, anesthesiologists traditionally have had limited participation in provider feedback initiatives, although they, as a specialty, are recognized as forerunners in patient safety emphasis. The Institute of Medicine (IOM) report, “To Err is Human,” recognized anesthesia care as one of the few health care disciplines that has taken effective actions to reduce medical error and improve patient safety [12,13]. In recognition, through the Anesthesia Quality Institute (AQI) and the National Anesthesia Clinical Outcomes Registry (NACOR), the American Society of Anesthesiologists (ASA) has created a framework for quality benchmarking and outcomes improvement in anesthesiology [14].

In surgical departments, provider feedback to modify health care practices is now accepted as standard practice. One of the first studies that assessed effectiveness of written feedback found that when providers received updates on their post-operative infection rates, along with guidelines for best practice, there was a statistically significant reduction in infection rates [15]. A twelve year Australian study found that notifying providers as a group of surgical site infection rates also resulted in a significant decline in infection incidence prior to discharge (4.7% to 1.2%; p < 0.001) [16]. Of note, this decline reversed entirely during a 15 month period when the provider notification system halted and was reproduced when it was re-instituted. Clearly, provider notification is an effective tool to reverse health practices with adverse effects and to reduce surgical site infection rates. Our hospital launched a study to assess the impact of notifying attending and resident surgeons and anesthesiologists jointly by letter of guideline violations on INF-1, INF-2, and INF-3 rates. The initiative began on March 1st 2010 and is on-going; our analysis had the following major objectives: (1) To create a multidisciplinary improvement process among surgeons and anesthesiologists to reduce surgical site infection rates, (2) To assess the effect on patient safety after notifying providers directly of their error on patient safety compliance, (3) To assess the internal financial ramifications of improved perioperative prophylactic antibiotic compliance.

## Methods

In order to improve compliance with SCIP perioperative antibiotic guidelines, our institution, a large tertiary academic medical center in New England, began to audit surgical cases for compliance and notified the anesthesia and/or surgical provider who ordered the incorrect antibiotic class, initiation timing or duration by letter stating the type (s) of error. We hypothesized that direct physician to physician feedback would result in significant compliance improvements. Provider notification and SCIP compliance data from March 1st 2010 to September 31st 2013 were analyzed to determine if error notification had an effect on prescriber behavior and if there was an improvement in compliance. Each quarter, approximately 10-12% of all inpatient surgical cases were randomly selected for audit by chart review. An average of 179 cases were audited each quarter out of 1,511 inpatient surgical cases. During a review meeting in the following quarter, cases with compliance errors were reviewed and providers were contacted by the antibiotic prophylaxis committee. Our intent was to involve both surgeons and anesthesiologists as part of the feedback committee and as recipients of feedback in order to break down inter-departmental barriers to communication. This was also seen as an opportunity to educate attending staff and residents involved in each specific clinical scenario.

In order to assess the effect of direct physician notification on patient safety compliance among anesthesiologists and surgeons, we trended provider non-compliance by department and quarter in Table 1. We examined the percentage of providers with repeat offenses in Table 2. We also contacted providers who had received >3 notification letters to assess why an individual would continue to make the same error after receiving feedback. The initiative was launched in Quarter 4 of 2010, and we compared data trends from this point on.

**Table 1:** **Surgical Care Improvement Project (SCIP) perioperative antibiotic delivery errors**

**SCIP error class**	**N (%)**	**2010**				**2011**				**2012**				**2013**	
		**Q1**	**Q2**	**Q3**	**Q4**	**Q1**	**Q2**	**Q3**	**Q4**	**Q1**	**Q2**	**Q3**	**Q4**	**Q1**	**Q2**
**INF-1:** Prophylactic antibiotic not delivered one hour prior to surgery start time	15 (21%)	0 (0%)	1 (7%)	2 (13%)	1 (7%)	2 (13%)	1 (7%)	1 (7%)	2 (13%)	1 (7%)	0 (0%)	0 (0%)	2 (13%)	0 (0%)	2 (13%)
**INF-2:** Incorrect prophylactic antibiotic selection for surgery type	16 (22%)	0 (0%)	3 (19%)	0 (0%)	4 (25%)	1 (6%)	2 (13%)	2 (13%)	1 (6%)	2 (13%)	0 (0%)	0 (0%)	1 (6%)	0 (0%)	0 (0%)
**INF-3:** Prophylactic antibiotics not discontinued within 24 hours after surgery end	40 (56%)	0 (0%)	4 (10%)	8 (20%)	4 (10%)	4 (10%)	4 (10%)	4 (10%)	2 (5%)	3 (8%)	1 (3%)	0 (0%)	2 (5%)	3 (8%)	2 (5%)

**Table 2:** **Provider non-compliance episodes by quarter**

**Department N (%)**	**2010**			**2011**				**2012**				**2013**		
	**Q2**	**Q3**	**Q4**	**Q1**	**Q2**	**Q3**	**Q4**	**Q1**	**Q2**	**Q3**	**Q4**	**Q1**	**Q2**	**Q3**
**Anesthesiology**	6	10	27	7	15	6	6	17	7	11	14	16	8	3
**Surgery**	12	8	33	19	15	21	14	26	14	18	40	39	13	5
**All**	17	18	60	26	30	27	20	43	21	29	54	55	21	8

Given the current emphasis on compliance- and outcomes-based reimbursement, we also evaluated the financial impact of improved antibiotic compliance in Table 3. All calculations were performed in Microsoft Excel 2004 for Macintosh (version 11.4.1, Microsoft Corporation, Redmond, WA). Temporal trends were evaluated for statistical significance with Chi-square and Fischer’s exact tests depending on sample size in SAS (version 9.4, SAS Corporation, Cary, NC). The final table column contains the net difference from FY2013 to FY2014. Of note, the bonus column does not contain the BCBS bonus as described above. A sensitivity analysis was performed per standard protocol assuming 20% variance to broaden our results to regions with differing health care costs. Complete financial data, including risk pool funds, net returns and compliance bonus data were available for inpatient perioperative antibiotic timing, selection, and duration for FY2013 and FY2014. The Centers for Medicare and Medicaid Services (CMS) risk pools included the Inpatient Quality Reporting (IQR) and Value Based Purchasing (VBP) programs. SCIP measure-specific financial data for the IQR program was calculated by dividing the total risk dollars associated with failure to report all measures by the number of category types included in IQR reporting. This amount was further divided by the number of measures included in the National Hospital Quality Measures (NHQMs) category for the respective calendar year and then once again by the number of SCIP measures reported in that calendar year to determine the cost associated with a particular SCIP measure. A similar revenue calculation was employed for the VBP program. The only difference between the two calculations was that the total risk dollars in the VBP program was multiplied by the weight of the clinical measures category for the respective VBP FY payment year, or 70% for FY13 and 45% for FY14. A detailed description of the risk pools is available upon request. Potential hospital bonuses from SCIP compliance were derived from the Mass Health site-specific bonus pool. Compliance with each measure was scored on a ten point system. Each point was equivalent to a 10% increase in bonus from the available bonus fund. Blue Cross Blue Shield (BCBS) also provided bonuses, but did not provide a breakdown of funds by measure. Therefore, all net totals are considered to be likely underestimates.

**Table 3:** **Providers with repeat offenses**

**Number of violations per physician**	**Anesthesiology**	**Surgery**	**All**
**2 offenses**	33	24	57
**3 offenses**	2	16	18
**4 offenses**	1	6	7
**5 offenses**	0	4	4
**>5 offenses**	1	5	6

## Results

Table 1 documents prescribing errors by INF guideline class and quarter. The percentage indicates the number of errors within the same class per quarter. Overall, the differences between individual quarters were not statistically significant; however, it is noted that the overall trend within each error class is downwards.

**Figure 1:**
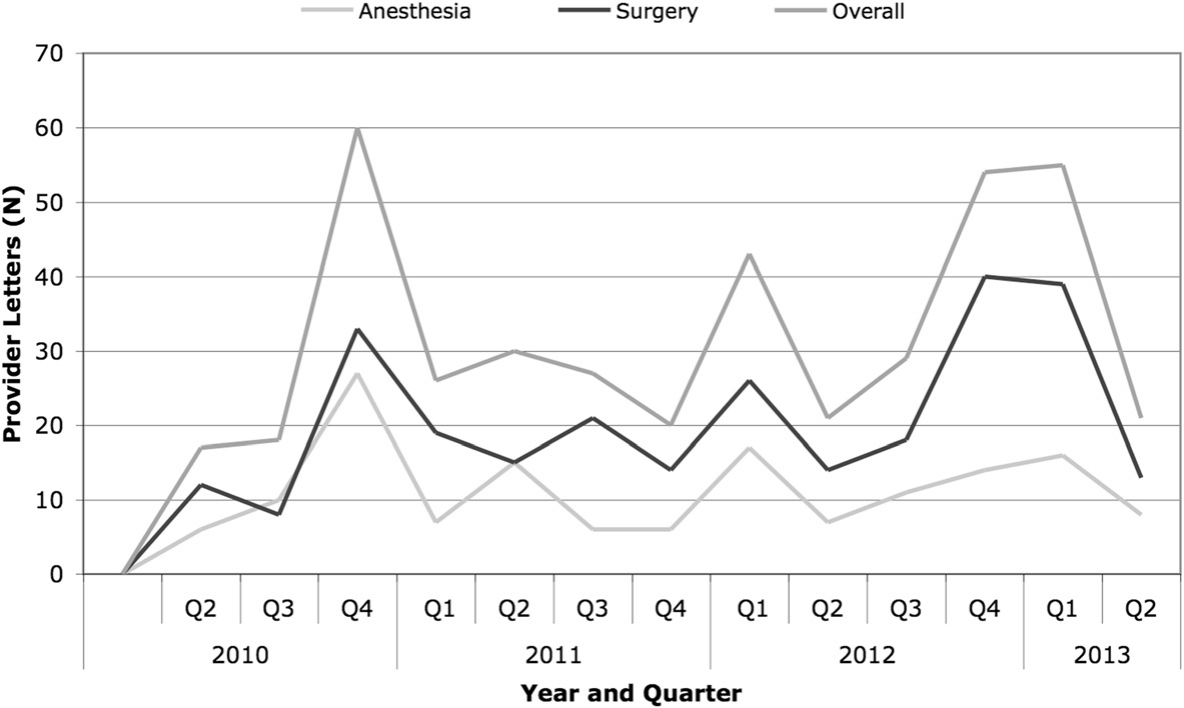
**Provider Notification Letters by Quarter and Department.**

Table 2 describes non-compliance episodes by department and by quarter. Figure 1 demonstrates these trends over the intervention time period. Overall, there was no significant decline over time in the number of letters sent. In two of three years with four quarters of available data, the peak quarter for notification included the months October-December. Table 3 describes providers with repeat INF guideline offenses by department and number of offenses. Only two anesthesia providers had greater than three offenses while 15 surgical providers fell within this category. Among providers who had received four or more error feedback letters, the most common reply was that they had delegated the antibiotic ordering responsibilities to a trainee or mid-level provider who was not aware of the guidelines.

In Table 3, FY2013 and FY2014 cost data are displayed by SCIP measure. By measure and fiscal year, Table 4 displays the risk pool amount, risk pool loss, potential bonus, actual bonus and annual net gain or loss. With regard to Inf-1, between money placed in the risk pool and insurance bonuses, there was a net return of $193,669 (+51%) in FY2013 and $221,048 (+63%) in FY 2014. Inf-2 compliance measures produced an additional $169,488 of hospital revenue in FY2013 and only $6,048 in FY2014. Inf-3 adherence resulted in a loss of $72,545 in FY2013 and in $18,000 the following year. In total, the hospital gained an additional $290,612 in 2011 and $209,096 in 2012 for improved compliance with three SCIP measures.

**Table 4:** **Peri-operative antibiotic compliance and cost savings to hospital**

	**Fiscal Year 2013 (2011 data)**					**Fiscal Year 2014 (2012 data)**					**∆ Net gain/loss FY 2013 to 2014**
	**Risk $** ^**[1]**^	**Risk pool loss**	**Potential bonus** ^**[2]**^	**Actual bonus**	**Net gain/loss**	**Risk $** ^**[3]**^	**Risk pool loss**	**Potential bonus**	**Actual bonus**	**Net gain/loss**	
	**(%∆;−/+20%)**	**(%∆;−/+20%)**	**(−/+20%)**	**(%∆;−/+20%)**	**(%∆;−/+20%)**	**(−/+20%)**	**(%∆;−/+20%)**	**(−/+20%)**	**(%∆;−/+20%)**	**(%∆;−/+20%)**	**(−/+20%)**
**INF-1 **	$139,087	-$48,364	$242,033	$242,033	$193,669	$103,465	-$27,000	$248,048	$248,048	$221,048	$27,379
	($111,270-$166,904)	(−35%;-$38,691-$58,037)	($193,626-$290,440)	(100%; $193,626-$290,440)	(51%; −$154,935-232,403)	($82,772-$124,158)	(−26%; −$32,400-$21,600)	($198,438-$297,658)	($198,438-$297,658)	(63%; $176,838-265,258)	(+12%; $21,903-$32,855)
**INF-2**	$139,087	-$72,545	$242,033	$242,033	$169,488	$103,465	-$18,000	$248,048	$24,048	$6,048	-$163,440
	($111,270-$166,904)	(−52%; $-87,054-$-58,036)	($193,626-$290,440)	(100%; $193,626-$290,440)	(44%; $135,590-$203,386)	($82,772-$124,158)	(−17%; −$21,600-$14,400)	($198,438-$297,658)	(10%; $19,238-$28,858)	(2%; $4,838-$7,258)	(−42%; −$196,128-$130,752)
**INF-3**	$139,087	-$72,545	$242,033	$0	-$72,545	$103,465	-$18,000	$248,048	$0	-$18,000	$54,545
	($111,270-$166,904)	(−52%; $-87,054-$-58,036)	($193,626-$290,440)	(0%; $0)	(−19%; −87,054-$58,036)	($82,772-$124,158)	(−17%; −$21,600-$14,400)	($198,438-$297,658)	(0%; $0)	(−5%; −$14,400-$21,600)	(+12%; $43,636-$65,454)
**Total**	$417,261	-$193,454	$726,099	$484,066	**$290, 612 **	$310,395	-$63,000	$744,144	$272,096	**$209,096 **	**-$81,516 **
	($333,809-$500,713)	(−$232,145-$154,763)	($580,879-$871,319)	($287,253-$580,879)	(+25%; $232,489-$372,474)	($248,316-$372,474)	(−$75,600-$50,400)	($595,315-$892,973)	($217,677-$326,515)	(+20%; $167,277-$250,915)	(−5%; $65,213-$97,819)

^[1]^Includes CMS Inpatient Quality Reporting (IQR) and Value Based Purchasing (VBP) risk pool data.

^[2]^Bonus to hospital from Mass Health’s Population Management Bonus Pool.

^[3]^Does not include National IPF quality bonus introduced in FY15 for SCIP3 only and BCBC bonus (data not made available to hospital by individual measure.

Bold text is indicated to highlight titles.

## Discussion

In summary, our institution sought to reduce surgical site infection rates and improve provider compliance by directly notifying providers of perioperative antibiotic dosing errors. Recent studies have demonstrated that notifying providers of short-comings or deviations from standard of care positively influence future behavior modification [17]. Overall, we were successful at improving SCIP compliance. Antibiotic selection (Inf-2) is a shared decision between anesthesiologists and surgeons, while the delivery time (Inf-1) is generally determined by the anesthesiologist at our institution. Antibiotic duration (Inf-3) is determined by the surgical team, typically the resident trainee placing postoperative orders. Given the overlap, we planned to include members of both departments in our feedback initiative with a common goal: to reduce surgical site infections among collective patients. We noted that repeat errors decreased dramatically among anesthesiologists who were audited, although the decline was less steep among surgical colleagues noted in Figure 1 and Table 2. When asked why they had received more than three notices, the most common reply was that a provider had delegated selection or duration duties to a mid-level provider or resident who was unfamiliar with the SCIP guidelines. It can be hypothesized that increased repeat errors among surgeons was also due to the less cohesive nature of one large department with both general and sub-specialty divisions that act independently.

Our analysis can be of value to both hospital administrators and health care providers because we are able to provide data on both SCIP antibiotic prophylaxis compliance and of the effect of provider notification on repeat errors. In addition, we provided data on the internal cost of non-compliance errors. To our surprise, our hospital had a significant increase in revenue generated as a result of increased compliance.

A 2012 Cochrane review noted that feedback is most effective when baseline compliance is low, which was not applicable in our analysis, and when it is provided by a colleague of equal or superior ranking [18]. The committee members that evaluated errors and provided feedback met this latter criterion. Additional characteristics of a successful prescriber audit are when the feedback is provided more than once, as we did with repeat offenses, when it is both verbal and written and when targets, guidelines and actions are described within the letter or commentary [19].

Overall, we were successful at improving overall perioperative antibiotic compliance and decreasing repeat errors. We believe that our provider notification initiative played a significant role in this institutional change [20]. As our revenue analysis illustrates, a low-cost peer-review audit intervention generated nearly $500,000 over two years for re-investment in patient safety initiatives. We infer that infection incidence fell as antibiotic compliance improved given the national data driving the creation of the Surgical Care Improvement Project [21,22].

Limitations of this analysis include the inability to survey providers as they received errors on their prior knowledge of the guidelines and why the error occurred. Recall bias could certainly be present among the providers who were surveyed 2–3 years after a primary error. We are also unable to retrospectively segregate providers who received feedback from those who did not and monitor the performance of both groups over time. The evidence and our experience support the causality of intervention and improved compliance. While only 10-12% cases were randomly selected for audit, the providers often participated in multiple cases each day. We had robust representation among surgical subspecialties and the anesthesia department.

Our analysis demonstrates that providers do modify critical clinical behaviors after notification of an error. It is our belief that the majority of providers desire this feedback in order to fulfill their oath and protect their patients to the best of their ability. Regardless of a provider’s feelings upon learning of a clinical error, peer audits and notification should become the norm in 21^st^ century health care.

## Conclusion

We piloted an intervention with minimal associated costs, focused on peer physician audits, and successfully reduced errors associated with SCIP perioperative antibiotic compliance. From our perspective, provider audit and subsequent notification of guideline non-compliance should be encouraged as standard practice to improve patient safety. The secondary benefit of increased revenue for re-investment in patient care serves as an additional incentive to adopt similar interventions.

## Abbreviations

SSI: Surgical site infection
SCIP: Surgical care improvement project
CMS: Centers for Medicare and Medicaid Services
CDC: Centers for Disease Control and Prevention
